# Florida Populations Most at Risk of Not Being Up to Date With Colorectal Cancer Screening

**DOI:** 10.5888/pcd15.170224

**Published:** 2018-05-31

**Authors:** Claudia X. Aguado Loi, Korede K. Adegoke, Clement K. Gwede, William M. Sappenfield, Carol A. Bryant

**Affiliations:** 1 College of Health and Natural Sciences, University of Tampa, Tampa, Florida; 2 Florida Prevention Research Center, College of Public Health, University of South Florida, Tampa, Florida; 3 College of Nursing and Public Health, Adelphi University, Garden City, New York; 4 H. Lee Moffitt Cancer Canter & Research Institute, Tampa, Florida

## Abstract

**Introduction:**

The purpose of this study was to examine the characteristics of populations at risk of not being up to date on colorectal cancer screening in Florida.

**Methods:**

We used Exhaustive Chi-squared Automatic Interaction Detection, a classification tree analysis, to identify subgroups not up to date with colorectal cancer screening using the 2013 Florida Behavioral Risk Factor Surveillance System. The data set was restricted to adults aged 50 to 75 years (n = 14,756).

**Results:**

Only 65.5% of the sample was up to date on colorectal cancer screening. Having no insurance and having a primary care provider were the most significant predictors of not being up to date on screening. The highest risk subgroups were 1) respondents with no insurance and no primary care provider, regardless of their employment status (screening rate, 12.1%–23.7%); 2) respondents with no insurance but had a primary care provider and were employed (screening rate, 32.3%); and 3) respondents with insurance, who were younger than 55 years, and who were current smokers (screening rate, 42.0%).

**Conclusion:**

Some populations in Florida are at high risk for not being up to date on colorectal cancer screening. To achieve Healthy People 2020 goals, interventions may need to be further tailored to target these subgroups.

## Introduction

Colorectal cancer (CRC) is the second leading cause of cancer death in the United States (1). CRC screening and early detection is an evidence-based strategy to reduce CRC morbidity and mortality (2,3). Yet, only 65% of US adults aged 50 to 75 years met the national CRC screening guidelines in 2012 (4). This disparity further widens in disadvantaged or ethnically or racially diverse groups (5). Thus, promotion of CRC screening, especially among at-risk populations, is a national priority.

The National Colorectal Cancer Roundtable (NCCRT) set a nationwide screening rate goal of “80% by 2018” (6), surpassing the Healthy People 2020 goal of 70.5% (7). Although Florida’s CRC screening rate (65.7%) ranks tenth of the 50 states, screening rates are below national goals (8,9). This new screening goal, adopted by more than 300 public and private groups, voluntary health care organizations, and advocacy groups including the American Cancer Society and the Southeastern Colorectal Cancer Consortium, represents progress in decreasing the national screening rates. This increase requires interventions designed for populations at risk for low CRC screening.

A description of populations at risk for low CRC screening is consistently evolving because of factors that include enactment of new policies (eg, Patient Protection and Affordable Care Act), population growth and diversity, and research and medical advancements. National studies have identified independent correlates (eg, access to health care, income level) of low CRC screening (10–12). Detecting the interaction (ie, combinations) of these correlates to identify populations at greatest risk for low CRC screening also has practical utility in planning targeted interventions; yet, this is rarely examined in the health disparity research. Using a tree classification analytical technique, Dominick and colleagues examined the interaction of these factors to identify high-risk subgroups with low CRC screening rates, using unweighted data from a national cancer health communication and information data set from 2007 (13). Since then, the US Preventive Services Task Force (USPSTF) updated the CRC screening guidelines in 2008 (14). Building on what is known in the literature and the latest USPSTF recommendations, we aimed to identify correlates and segments of populations at risk for not being up to date with CRC screening, using data from a large, statewide population-based survey in Florida.

## Methods

### Data source and design

We analyzed data from the 2013 Florida Behavioral Risk Factor Surveillance System (BRFSS). This state-based annual telephone surveillance system is designed to collect data on individual risk behaviors and health practices related to the leading causes of illness and death in the United States. The Centers for Disease Control and Prevention (CDC) provides financial and technical support to all 50 states, as well as the District of Columbia and 3 US territories, to conduct BRFSS. Survey information is generally used for health planning, program evaluation, and monitoring health objectives (8).

The 2013 Florida BRFSS used disproportionate stratified sampling to collect data from respondents aged 18 years or older who resided in a Florida household (N = 34,186). The survey response rate was 35.2%. In disproportionate stratified sampling, telephone numbers were drawn from 2 sets of telephone number blocks, and one adult was randomly selected from eligible households. The ranking weights provided by the CDC were applied to the data to improve representativeness to the Florida adult population. Data were weighted to the respondent’s probability of selection by county, as well as by age, sex, marital status, race/ethnicity, and education level.

### Participants

Our analysis sample included adults within the recommended age for CRC screening, aged 50 to 75 years (n = 14,756). Respondents were excluded from the analysis if they had missing responses for any of the measures of interest (10.8%) except for income because of the high prevalence of missing data (9.5%). This study received institutional review board approval from the University of South Florida and the Florida Department of Health.

### Variables

The outcome, being up-to-date with CRC screening, was based on 2008 USPSTF recommendations (14). Respondents who met any one of the following criteria were classified as being up to date: 1) self-report of fecal occult blood test (FOBT) during the past year, 2) sigmoidoscopy in the past 5 years and FOBT in the past 3 years, or 3) colonoscopy in the past 10 years.

Thirteen independent variables were selected to examine adherence to CRC screening guidelines. Selection of these variables in the data set was based on recommendations from an academic and research team with expertise in CRC screening health disparities and the published literature (10–12). These variables were sociodemographic characteristics (age [50–54 y, 55–59 y, 60–64 y, 65–69 y, 70–75 y], sex [male, female], race [non-Hispanic white, non-Hispanic black, other], ethnicity [Hispanic, non-Hispanic], marital status [married/partnered, divorced/separated/widowed, never married], educational level [<high school graduate, high school graduate or general educational development, some college or technical school, college graduate], income level [$0–$14,999; $15,000–$34,999; $35,000–$49,999; $50,000–$74,999; ≥$75,000], employment status [employed, unemployed, retired, unable to work, student/homemaker]), indicators of access to care (have at least one primary care provider, do not have a primary care provider), and health status or behavior (body mass index [not overweight, overweight, obese], smoking status [current, former, never], general health status [excellent, good/very good, fair/poor]). Age was initially examined as a continuous variable but was later categorized into 5 groups on the basis of natural cut-points identified during the analysis and our CRC experts’ recommendation.

### Statistical analysis

We used descriptive statistics to describe the study population and χ^2^ tests to examine differences in sample characteristics by CRC status.

We constructed a classification tree using Exhaustive Chi-squared Automatic Interaction Detection (E-CHAID), a statistical procedure commonly applied in marketing, using SPSS version 24 (SPSS Institute, Inc). E-CHAID uses a multivariate, algorithm-based method to classify combinations of variables on the basis of their correlation with an outcome of interest (15,16). The procedure makes no assumptions about the probability distributions of the variables being assessed. Statistically significant subgroups, or segments, of the population are generated and presented in a decision tree (16–19). This method has been applied in studies of breast cancer screening (16,17) and to examine characteristics of low CRC screening in a national data set (14).

E-CHAID systematically split the sample from the dependent variable root node through a series of parent and child nodes to the final set of terminal nodes (19–21). Variables that produced maximum homogeneity of individuals with the outcomes of interest within the node were chosen to form the tree. Our a priori criteria, as in the case of those of other studies that assessed CRC screening (13,17), set the tree to grow up to 7 levels. Splits could occur only in a parent node with 5% or more of the total sample; each child node had to contain at least 2.5% of the total sample. Our sample population adjusted to approximately 4.7 million individuals after applying the frequency weight. Each parent node and child node included at least 235,477 and 117,738 individuals, respectively. We applied a 10-fold cross-validation to assess the tree structure’s predictability performance, which was 73.2% (22). Segments below the average CRC screening rates in Florida were defined as at-risk subgroups. Segments in the lowest CRC screening rate quartile were defined as high-risk subgroups.

## Results

The up-to-date CRC screening rate was 65.5% (Table 1). Significant associations were found for all study variables by CRC status (yes or no). Compared with individuals who were not up to date on CRC screening, individuals who were up to date were older, were female, were college graduates, had higher incomes, and were married or partnered (*P* < .001). Compared with individuals who were not up to date on screening, more people who were up to date were non-Hispanic white, non-Hispanic, and insured, and more also had a primary care provider (*P* < .001).

**Table 1:** Weighted Distribution of Respondents’ Characteristics, by Colorectal Cancer Screening Status, Florida BRFSS, 2013^a^

Characteristic	Overall % (N = 14,756)	Up to Date With Colorectal Cancer Screening^b^	
		% Yes (n = 10,292)	% No (n = 4,464)
**Age, y**			
50–54	24.7	17.2	38.8
55–59	20.5	18.8	23.7
60–64	19.9	20.8	18.0
65–69	17.5	21.5	9.7
70–75	17.5	21.6	9.8
**Sex**			
Female	52.5	52.7	52.0
Male	47.5	47.3	48.0
**Race**			
White, non-Hispanic	69.5	71.6	65.4
Black, non-Hispanic	10.9	10.9	10.7
Other	19.6	17.4	23.8
**Ethnicity**			
Hispanic	17.0	15.1	20.6
Non-Hispanic	83.0	84.9	79.4
**Marital status**			
Married/partnered	63.8	67.9	56.1
Divorced/separated/widowed	30.0	27.4	35.3
Never married	6.2	4.7	8.7
**Educational level**			
Less than high school graduate	13.3	11.2	17.3
High school graduate or GED	29.3	27.7	32.2
Some college or technical school	32.5	33.5	30.5
College graduate	24.9	27.5	20.0
**Income level, $**			
0–14,999	11.9	10.0	15.7
15,000–34,999	15.2	13.6	18.2
35,000–49,999	11.1	10.0	13.3
50,000–74,999	14.0	14.0	14.0
≥75,000	38.3	43.1	29.2
Missing/refused	9.5	9.4	9.7
**Employment status**			
Employed	44.9	40.5	53.3
Unemployed	6.9	4.7	11.3
Retired	31.8	39.3	17.6
Unable to work	10.7	10.6	10.8
Student/homemaker	5.7	5.0	7.0
**Health care coverage**			
Yes	85.3	93.9	68.9
No	14.7	6.1	31.1
**Have primary care provider**			
Yes, at least one	85.6	93.6	70.4
No	14.4	6.4	29.6
**Body mass index category (kg/m^2^)**			
Not overweight (≤24.9)	29.1	27.0	33.0
Overweight (25.0–29.9)	40.9	40.6	41.6
Obese (≥30.0)	30.0	32.5	25.4
**Smoking status**			
Current	17.2	13.7	23.9
Former	38.7	40.1	36.2
Never	44.1	46.3	39.9
**General health status**			
Excellent	17.0	14.8	21.3
Good/very good	59.2	61.6	54.5
Fair/Poor	23.8	23.6	24.2

Abbreviations: BRFSS, Behavioral Risk Factor Surveillance System; GED, general educational development.

a Weighted to BRFSS complex survey sampling weights.

b All comparisons of respondents’ characteristics by up-to-date status of colorectal cancer screening were significant at *P* < .001.

Of all 13 variables included in the classification tree analysis (CTA) model, whose initial splitting variable (ie, the parent node for which all subsequent subgroups were formed) was having health insurance, E-CHAID dropped income, race, and ethnicity (Figure). The uninsured population constituted 14.7% of our weighted study population and had a CRC screening rate of 27.2%. The up-to-date screening rate was almost 2.7 times as high among the insured population (72.1%). The uninsured population with a primary care provider had a CRC screening rate of 40.4% versus 16.8% among the uninsured who did not have a primary care provider. Screening rates increased overall with age, and further splits occurred among age categories. Among those aged 50 to 54 years, smoking status was the splitting variable. For individuals aged 55 to 59 years, marital status was the splitting variable. For populations aged 60 to 64 years, 65 to 69 years, and 70 to 75 years, there were further divisions by body mass index (BMI), general health status, and educational level. Sex was the last splitting variable for CRC screening in the analysis.

**Figure:**
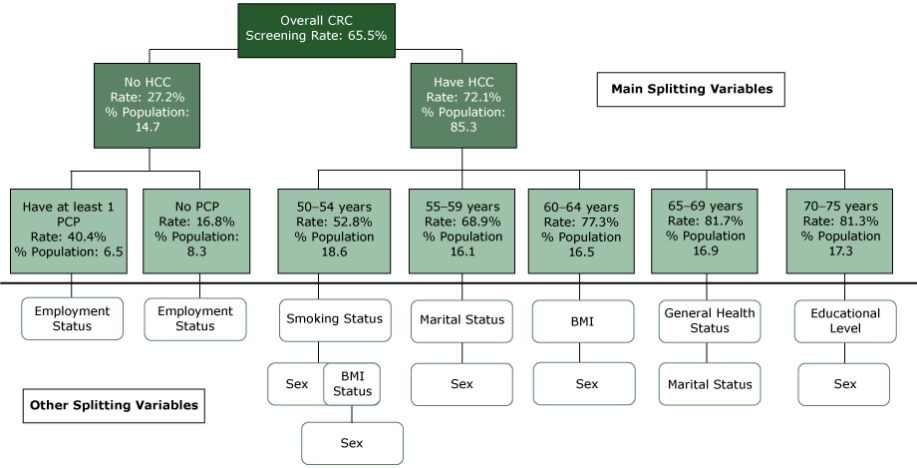
Classification tree diagram for up-to-date status for CRC screening among adults aged 50 to 75 years, Florida Behavioral Risk Factor Surveillance System, 2013. Abbreviations: % Population, % of total weighted sample size in each node; BMI, body mass index; CRC, colorectal cancer; HCC, health care coverage; PCP, primary care provider.

There were 48 nodes with 28 terminal nodes or distinct subgroups of the study population identified by CTA (Table 2). Up-to-date CRC screening rates ranged from 12.1% to 88.3% for the subgroups. The 25% to 75% interquartile screening rates ranged from 41.8% to 65.8%. The lowest quartile of nodes had screening rates ranging from 13.3% to 48.1% and accounted for 45.9% of the population not up to date with CRC screening. The highest quartile had rates from 81.6% to 88.3% and accounted for 10.0% of the not-up-to-date population. There were 11 segments of the population with screening rates below Florida’s average rate of 65.5%. Node 27 (individuals with no insurance; no primary care provider and were either employed or students/homemakers) had the lowest screening rate (12.1%). This rate was followed by node 26 (individuals with no insurance; with no primary care provider; and who were unemployed, retired, or unable to work) and node 25 (individuals with no insurance; with a primary care provider; and who were employed or students/homemakers) with 23.7% and 32.3% screening rates, respectively. Individuals with insurance, who were aged 50 to 54 years, and who were current smokers (node 10) were another high-risk group (screening rate, 42.0%). Those who were insured, were aged 70 to 75 years, and who were at least a college graduate (node 23) had the highest screening rate (88.3%).

**Table 2:** Results of Classification Tree Analysis for Colorectal Cancer Screening, Florida BRFSS, 2013^a^

Node, No.	Description	Screening Rate, %	Target Population, No.^b^	% of Target Population
**Nodes below the average colorectal cancer screening rate in Florida **				
27	HCC = no; PCP = no; employment status = employed, student/homemaker	12.1	203,948	12.5
26	HCC = no; PCP = no; employment status = unemployed, retired, unable to work	23.7	119,446	7.3
25	HCC = no; PCP = yes; employment status = employed	32.3	96,821	6.0
10	HCC = yes; aged = 50–54 y; smoking status = current smoker	42.0	96,247	5.9
28	HCC = yes; aged = 50–54 y; smoking status = former or current smoker; sex = male	43.1	76,548	4.7
24	HCC = no; PCP = yes; employment status = unable to work, retired, unemployed, student/homemaker	47.5	85,554	5.3
29	HCC = yes; aged = 50–54 y; smoking status = former or current smoker; sex = female	48.1	69,033	4.2
45	HCC = yes; aged = 50–54 y; smoking status = never smoked; BMI = not overweight, overweight; sex = female	52.3	87,292	5.4
32	HCC = yes; aged = 50–54 y, 55–59 y; marital status: separated/divorced, married, never married; sex = male	54.1	59,837	3.7
33	HCC = yes; aged = 50–54 y, 55–59 y; marital status: previously married, never married; sex = female	59.9	54,490	3.3
44	HCC = yes; aged = 50–54 y; smoking status = never smoked; BMI = not overweight, overweight; sex = male	62.4	46,009	2.8
**Nodes above the average colorectal cancer screening rate in Florida **				
35	HCC = yes; aged = 50–54 y, 55–59 y; marital status: married/partnered; sex = female	70.1	76,775	4.7
16	HCC = yes; aged = 55–59 60–64 y; BMI = not overweight	70.9	62,110	3.8
31	HCC = yes; aged = 50–54 y; sex = female; smoking status = never smoked; BMI = obese	72.3	37,681	2.3
37	HCC = yes; aged = 55–59, 60–64 y; BMI = overweight, sex = female	73.2	43,614	2.7
42	HCC = yes; aged = 70–75 y; educational Level = high school or GED, some college or technical school; sex = male	75.8	30,427	1.9
19	HCC = yes; aged = 60–64 y, 65–69 y; general health = excellent	75.8	46,229	2.8
21	HCC = yes; aged = 70–75 y; educational Level = less than high school or GED, high school or GED	76.4	61,226	3.8
40	HCC = yes; aged = 60–64 y, 65–69 y; general health = fair/good, good/very good, marital status: previously married	78.8	33,638	2.1
36	HCC = yes; aged = 55–59, 60–64 y; BMI = overweight, sex = Male	80.1	30,410	1.9
34	HCC = yes; aged = 50–54 y, 55–59 y; marital status: married/partnered; sex = male	81.2	43,880	2.7
38	HCC = yes; aged = 55–59, 60–64 y; BMI = obese; sex = male	81.6	21,747	1.3
47	HCC = yes; aged = 60–64 y, 65–69 y; general health = fair/good, good/very good, marital status: never married, married/partnered; smoking status = never smoked	82.3	33,052	2.0
20	HCC = yes; aged = 70–75 y; educational Level = less than high school	83.0	21,465	1.3
43	HCC = yes; aged = 70–75 y; educational Level = high school or GED, some college or technical school; sex = female	85.4	18,700	1.1
39	HCC = yes; aged = 55–59, 60–64 y; BMI = obese; sex = female	86.0	17,860	1.1
46	HCC = yes; aged = 60–64 y, 65–69 y; general health = fair/good, good/very good, marital status: never married, married/partnered; smoking status = current smoker, former smoker	87.4	32,543	2.0
23	HCC = yes; aged = 70–75 y; educational Level = college graduate	88.3	20,270	1.2

Abbreviations: BMI, body mass index; BRFSS, Behavioral Risk Factor Surveillance System; GED, general educational development; HCC, health care coverage; PCP, primary care provider;

a The average colorectal screening rates in Florida was 65.5% (unweighted no. = 14,756; adults aged 50–75 y).

b Weighted number of participants not up to-date with CRC screening.

## Discussion

Florida’s diversity uniquely positions the state to examine CRC screening and cancer health disparities. Cancer is the leading cause of death among its residents, and the state has the second highest cancer prevalence in the nation (23). In this study, we used CTA to identify the characteristics of populations at high risk of not being up to date with CRC screening in Florida. We found that insurance status and primary care provider status were the strongest predictors of CRC screening. Our study adds to the literature by isolating groups of variables that interact to define high-risk segments of the population specific to low CRC up-to-date status in Florida. In other words, our study identified statistically significant, distinct segments of the population with homogenous characteristics associated with the dependent outcome (not being up to date with CRC screening). Individuals who had no insurance and no primary care provider, regardless of their employment status, had the lowest screening rate (12.1%– 23.7%). Other high-risk subgroups identified were 1) employed individuals who had a primary care provider but no insurance and 2) individuals who were insured and younger than 55 years.

Our findings are distinct from those of most previous studies on CRC screening, because those studies only investigated individual risk factors for low screening without examining interactions that existed between them (11,12). In our study, we not only identified several interaction terms that predict CRC screening, but also found well-known factors, consistent with the literature, that are associated with low screening. These factors include lack of insurance, lack of primary care provider, low levels of education, and younger age. Few studies have used CTA to examine sociodemographic factors that influence CRC screening (13,18) and screening for other types of cancer (16,17,24).

We identified insurance status as the primary splitting variable. A study that used CTA to assess breast cancer screening also found insurance status and primary care provider status to be among the strongest predictors of screening (17). Access to health care is a commonly cited barrier to CRC screening (4,10,13,18). Although our data set may not reflect the influence of the recently implemented Patient Protection and Affordable Care Act, future data sets may indicate a reduction in this disparity. Race, ethnicity, and income variables were not significantly associated with our study outcome variable and were therefore dropped when running the CTA. These results differ from previous findings on health screening that identified income as an important splitting variable (16,17,24). In a study by Dominick et al that used CTA, income was a significant but minor splitting variable in predicting CRC screening. Findings from a national study, which used data from the 2007 Health Information National Trends Survey, also did not identify race/ethnicity as significant variables in predicting CRC screening (13). The literature on CRC screening health disparities often emphasizes disparities by race/ethnicity (10). However, more recent literature found this association to dissipate after controlling for differences based on socioeconomic status. For example, Burgess et al demonstrated that observed racial/ethnic disparities in CRC screening were no longer present after controlling for demographic and health factors (25). Thus, our insignificant finding suggests that other factors such as having insurance coverage and a health care provider are greater drivers in predicting CRC screening in Florida than disparities in household income and race/ethnicity.

The terminal node subgroups with the lowest CRC screening rates included respondents who not only lacked insurance, but also had no regular primary care provider. This finding is consistent with those of previous research that identified primary care provider status as a key splitting variable in screening (13,18,24). Similar to our findings, having a primary care provider was the second most important determinant (first splitting variable) of screening among the segment with no health insurance (13,23). However, in the Gjelsvik et al CTA study on mammography use among US women, “having a primary care provider” was the primary splitting variable (17). Even though some findings from previous CTA studies on screening are similar to ours, making comparisons is difficult because of differences in outcomes or how they were defined, for example, and how results are dependent on the number, type, and coding of variables included in the model. For instance, Dominick et al found that the subgroup that was least adherent to screening included individuals who avoided doctors not for fear of illness or death, were younger (50–64 y), and did not have a regular health care provider (CRC screening rate, 25.8%). We also observed that younger individuals eligible for CRC screening, particularly those younger than 60 years, had lower adherence rates. In contrast, we coded age differently, using 5-year intervals based on the natural split created by E-CHAID when the variable was initially examined as a continuous one. Also, we did not have any variable that assessed doctor avoidance in our data set.

Attention to both at-risk subgroups and high-adherent subgroups is necessary to achieve CRC screening rates that meet or surpass national goals. Our findings show that segments of the population with the highest screening rates represent less than two-thirds of the population, which only accounted for 10% of the not-up-to-date population. Although intervention efforts may consider outreach to nonadherent individuals from these segments, these subgroups may, despite access to health care, encounter impediments that are hard to modify (eg, strong beliefs and attitudes). Segments of the population below the Florida CRC screening average represent more than one-third of the population and slightly less than half of the nonadherent CRC screening population. Investing public health efforts among these segments holds the best promise to increase screening rates. These segments include subgroups without health care insurance, without a primary care provider, or both. Without policies to improve access to screening completion (including referral and follow-up screening services) and providers, achieving national goals is unattainable. Likewise, providing outreach to segments that include younger individuals with health care access who meet screening guidelines is also necessary. This subgroup, coined the “unworried well” by the American Cancer Society, includes individuals that may not consider CRC screening as a priority health concern (26). In summary, subgroups with high CRC screening rates represent the largest proportion of the population but are insufficient in size to meet national goals without providing outreach to populations at greatest risk whose screening rates are below state and national averages.

This study has limitations. First, the Florida BRFSS has a low response rate, which may result in nonresponse bias. Nonresponders who refused to participate in the survey may differ from the respondents and the entire population. Second, 10.8% of the total observations for individuals aged 50 to 75 years were deleted due to missing data on variables of interest. As with the case of nonresponders, participants excluded from the analysis may differ from those included. If the characteristics of nonrespondents or participants with missing data are distinct from the actual target population, screening prevalence may be underestimated or overestimated, making our results less generalizable. Third, the outcome of the study was self-reported, which may result in recall bias (especially with the timing of the last screening tests) and social desirability bias. Fourth, the outcome was derived from questions that assessed the use of FOBT, sigmoidoscopy, and colonoscopy, in general. Some respondents may have used these tests for diagnostic purposes rather than screening. Research shows that national surveys overestimate the true prevalence of screening (27). This meta-analytic study of validation studies examining the accuracy of self-reported cancer-screening histories found sensitivity of approximately 0.80 for colorectal screening histories; even lower estimates were found in samples with predominantly black and Hispanic participants compared with samples with predominantly white participants. These biases from the use of a self-report measure must be considered when interpreting our results, bearing in mind that the true rates may be far below the estimated values. Finally, these results are dependent on the variables included on the 2013 Florida BRFSS. Variables not collected in the 2013 data set but that may need further investigation include provider recommendation, doctor avoidance, fear of CRC, and family history of CRC (13,18,28). Provider recommendation is a strong predictor of CRC screening (27). Studies indicate that compliance with CRC screening guidelines is improved when providers discuss options and make specific screening test recommendations. As in previous CTA screening studies, we could not investigate the effect of provider recommendation in defining populations at risk for low screening, but we could examine the interactive effects of whether they had a provider.

Our study has several strengths. The data were weighted to improve the generalizability of our results to Florida residents aged 50 to 75 years. To the best of our knowledge, this is the first study conducted using Florida data to identify subgroups that share the same patterns of characteristics in terms of not being up to date with CRC screening. Studies indicate that CTA is a powerful decision-making tool (29) and a promising strategy to tailor interventions to population subgroups at high risk (30). Compared with cluster analysis or logistic regression analysis, the visual image of a hierarchical tree structure provides benefit to CRC practitioners, researchers, community partners and policy makers who are involved in deciding the priority populations in which to improve CRC screening rates.

On the basis of this study’s strengths, the Florida Prevention Research Center presented results to stakeholders at community and national organizations including the American Cancer Society, NCCRT, statewide health departments, and local health and employee coalitions to facilitate policy and program changes. Because of the visual ease of understanding, the tree structure enhanced dialog among stakeholders because its form as a decision tree (or an organizational chart) has the most influential variables on top. It also gave an estimate of the population size in each high-risk subgroup in addition to the subgroup characteristics. This allowed for prioritization in the selection of target population and estimation of population that may be reached, the next step in this research.

As we approach the deadline of “80% by 2018” CRC screening rates (6), only 65.5% of Floridians are up to date with CRC screening, with rates as low as 12% among some subgroups. To improve the CRC rates in Florida and be able to achieve the NCCRT/Healthy People 2020 goals, a focus on high-risk segments is required. Individuals with no health insurance and no primary care provider is well known as a high-risk group, but attention to segments with a primary care provider and who are younger than 55 years may be overlooked. Using best practices when working with communities and other stakeholders in CRC screening, information gained from this analysis can be incorporated to narrow decisions to adapt, develop, or implement evidence-based interventions to improve CRC screening rates among high-risk subgroups.
